# The Individual Work Performance Questionnaire: Psychometric Properties of the Italian Version

**DOI:** 10.3390/ejihpe14010004

**Published:** 2023-12-22

**Authors:** Silvia Platania, Martina Morando, Stefania Valeria Gruttadauria, Linda Koopmans

**Affiliations:** 1 Section Psychology, Department of Educational Sciences, University of Catania, 95124 Catania, Italy; martina.morando@phd.unict.it (M.M.); stefigrutt@gmail.com (S.V.G.); 2 TNO Healthy Living and Work, Department of Sustainable Productivity and Employability, 2333 BE Leiden, The Netherlands; linda.koopmans@tno.nl

**Keywords:** work, performance, validation, psychometric properties, satisfaction

## Abstract

Individual work performance can be defined as individual behaviour capable of generating value and a competitive advantage for the organization. Furthermore, this construct is linked to other fundamental variables that constitute worker well-being, such as job satisfaction and engagement. Although important, a complete measure of individual work performance is still lacking in the Italian context. The objective of this work is to validate the Individual Work Performance Questionnaire (IWPQ) within the Italian organisational context. The IWPQ is a multi-dimensional construct consisting of task performance, contextual performance, and counterproductive work behavior. To investigate the psychometric properties of the Italian IWPQ, 1053 participants were enrolled, whose ages ranged between 19 and 69 years. EFA, CFA, and MCFA analyses were performed to test the structural factors of the IWPQ. The results supported the validity of the IWPQ in the Italian context; the final structure consisted of 17 items. Multigroup confirmatory factor analysis showed that the factor solution was invariant across both gender and occupational categories and found evidence of metric, uniqueness, scalar, and structural invariance. Convergent validity was also tested and demonstrated. Adequate studies on the importance of individual performance can be used to better understand and distinguish the different components affecting performance.

## 1. Introduction

In modern organisations, the evaluation of individual work performance plays a critical role in measuring the contribution of individuals to the success of the company. Indeed, work performance can be defined as the individual behaviour capable of generating value and competitive advantage for the organisation [1,2,3]. Work performance represents a key and founding variable in almost all areas of management and organisational behaviour. In this sense, performance management is thus a key process for optimising human resources and achieving organisational goals. For this process to be effective, however, it is essential to have a comprehensive and complete definition of the nature of job performance and to dispose of reliable and valid instruments to accurately assess processes and behaviours associated with employee performance [4].

Over the years, several empirical contributions have found that Individual Work Performance (IWP), understood at both the individual and collective level, can be interpreted and considered in terms of its broader meaning of organisational performance [5,6]. This underlines how different the core components of work performance are and that the instruments adopted and chosen for their measurement must necessarily adapt to this new and more precise interpretation.

Several studies analyse the importance of IWP by examining it as a relevant factor in increasing corporate profit, team performance, and ensuring the organization’s competitiveness in the business world. Campbell and Wiernik [4], for example, claimed the close link between IWP and group performance, with clear and direct results and effects on sense of unity, organisation, economic performance, and gross domestic product (GDP). Therefore, it is not an exaggeration to say that IWP represents the pivot on which organisations thrive. Indeed, Armstrong [7] pointed out in one of his works that ensuring the achievement of a higher level of IWP is one of the key responsibilities that managers of organisations assume to ensure success and competitive advantage. According to Koopmans et al. [8], moreover, IWP can be viewed as a key indicator for team and organisational performance and consequently has an impact on the organisation’s productivity and competitive ability.

Several theoretical models and conceptual frameworks were developed by research and literature in an attempt to capture key aspects and factors of performance. Among the existing theoretical models, Motowidio [9] frames work performance as based on specific behaviours that occur within a defined time period. These behaviours constitute the expected value and expectations for the organisation and represent the way in which it discriminates between behavioural patterns performed by different workers or by the same worker but at different times. The crucial distinction is based on the likelihood that the set of these behaviours could contribute to or threaten organisational effectiveness [4,9,10]. Therefore, performance embraces only those behaviours that have the potential to positively influence the achievement of the goals set by the organisation [11]. Campbell’s [12], a more recent contribution, is instead related to the formulation of a multifactorial model for interpreting work performance. He identifies eight factors: (1) task-specific competence; (2) competence in non-job-specific tasks; (3) written and oral communication; (4) demonstration of commitment; (5) personal discipline; (6) facilitation of team and peer performance; (7) supervision; and (8) management and administration. This model explains the complexity and multi-perspective nature of the performance construct, influenced by both internal or dispositional and external or situational factors. Other empirical evidence also showed the influence of these factors, e.g., among the internal ones: personality [13], motivation [14], job satisfaction [15], and among the external ones’ organisational constraints [16], leadership [17], or the work environment [18].

Within this framework, other models attempted to interpret performance, always pointing to its complex and multi-perspective nature. An example is given by Sonnentag and Frese [19], who in 2002 developed a model in which task-oriented work activities (which are adapted to the technical core of the organisation) and context-oriented performance (non-technical work activities that are rooted in the social, organisational and social and psychological context) were distinguished. Other empirical contributions [20,21], based on the Abramis [22] model, advanced and developed further models, strengthening knowledge on the topic. Abramis [22] defined work performance as the ability of workers to successfully perform their tasks and make a positive contribution to the work environment. In his model, he identified three dimensions: technical performance (the ability to manage tasks, good decision-making, and correct execution of tasks), social performance (good teamwork, the ability to manage conflicts appropriately), and presence (no tardiness or absence at work). Peiró et al. [21], based on this model, developed a model and measurement instrument to assess the work performance of workers by considering six tasks: making decisions, performing without making mistakes, achieving goals, commitment, taking initiative, and taking responsibility.

Although the international literature considers the evaluation of organisational performance a necessary activity to foster good performance in organisations, in Italy the legislation has only recently explicitly addressed this topic (d.lgs. 150/2009; d.lgs. 74/2017), and even from a scientific point of view, there are few existing measurement scales. Several scales have been developed to measure the dimensions of the IWP, among which we recall Williams and Anderson [23], who developed a short form and a brief task performance scale; the contextual performance evaluation developed by Podsakoff and MacKenzie [24]; and Van Scotter and Motowidlo [25]. Despite this, in the Italian context, there is still no use of a specific measure that measures individual work performance. To the best of our knowledge, only recently did Di Fabio and Svicker [26] develop a short and single-factor version of the Self-rated Job Performance Scale. Therefore, in view of these elements, the aim of the present work was to validate and adapt the Individual Work Performance Questionnaire in the Italian context, providing, from a theoretical and applicative point of view, a broad instrument including various performance factors, as described and meant by the literature. Currently, the Italian labour situation is affected by several issues: political, cultural, and profit issues that disincentivize companies from investing in human resources. The employment needs of organisations are mostly underestimated. As a result of such organisational policies, fatigued workers are produced with an IWP that does not live up to its true potential [27,28]. Organizations aim to maximise profits with minimal expenditure while ignoring the needs and requirements of employees. The importance and relevance of applying such a tool could give Italian organisations the opportunity to evaluate not only IWP but also organisational performance. Such a new view can help organisations see the effectiveness of an investment in IWP and employee well-being. By changing organisational policy, it is hypothesised that greater productivity could be realised not only for the organisation itself but also for the Italian working world. In this regard, it is important to check whether the instrument performs equally well in terms of both gender and different categories of workers.

## 2. The Individual Work Performance Questionnaire

Several instruments have been developed in the international landscape for an adequate measurement of the performance construct. However, those existing instruments presented limitations over time, as they were not able to measure all relevant aspects of individual work performance [29] or were developed for specific worker populations [10]. Overcoming the limitations of these existing instruments, the Individual Work Performance Questionnaire (IWPQ) was developed [10], which, for the Italian scenario, seems to be a valid alternative to identify and assess individual performance. Having a valid and useful tool available to Italian organisations to survey individual performance will only serve as an incentive to take employee and group well-being into greater consideration.

Based on a systematic review [8], the IWPQ consists of three main domains: task performance, contextual performance, and counterproductive work behaviour.

Task performance can be defined as the set of behaviours that contribute to the production of goods or services. It represents the competence or ability to perform the main or central tasks of the job [8] and is related to behaviours prescribed by the role and included in the role description [30]. This dimension consists of completing work tasks, maintaining up-to-date knowledge, working accurately and neatly, and planning, organising, and solving problems [8].

The second dimension is contextual performance, also referred to as organisational citizenship behaviour (OCB). It can be defined as “behaviour that contributes to the organisation’s goals by contributing to its social and psychological environment” [31] (pp. 67–68). This dimension includes extra-role behaviours and actions in support of the organisation, including initiative, proactivity, and cooperation [8]. The main distinction with task performance is that these behaviours are not embedded in the role description but rather are oriented towards promoting the effective functioning of the organisation, with an unnecessary direct effect on employee productivity [32]. Typical examples of contextual performance are: influencing others to engage in organisational citizenship behaviours; increasing personal willingness to work; or showing positive influence towards human resources in the organisation.

Finally, the third dimension is counterproductive work behaviour, defined as “voluntary behaviour that damages the well-being of the organization” [31] (p. 69). These behaviours have a negative value for the organisation as a whole as they conflict with organisational goals and effectiveness [9,33]. Counterproductive behaviours include complaining, off-task behaviour, presenteeism, abuse of privileges, misuse of information, time, and resources, unsafe behaviour and poor work quality [8]. Within the domain of counterproductive behaviours, a two-dimensional structure can be detected, which is also reflected at the level of consequences: there are indeed deviant behaviours related to people (e.g., gossiping about colleagues and differences) and behaviours related to organisations (e.g., absenteeism) [34,35,36].

Although the three dimensions have a relationship with each other, the literature suggests the importance of analysing each dimension separately, as each dimension has its own identity and domain and takes into account peculiar and distinct aspects of performance that need to be assessed for full understanding and measurement [37].

## 3. The Aim of This Study

Although individual work performance (IWP) is a topic of great importance in the organisational context, there is still little attention paid to the importance of defining it conceptually and measuring it in the Italian context. Work performance is often regarded as a necessary outcome variable for the survival of companies and for the well-being and satisfaction of workers, but equally, it is neglected, especially in terms of its proper conceptualisation and measurement. Developing and validating instruments and then comparing them in different European countries and testing their validity and standardisation can help research and related work practises start an important debate on the importance of work performance [38,39]. The original Dutch version of the IWPQ [10] has been translated into English-American [40], Spanish [39], and Swedish [38], and various translations in other European languages are underway. In the logic of comparison and adaptation of the scale in the Italian organisational context, it is considered important to conduct a cross-cultural comparison study with the results of the Dutch version in which the IWPQ was developed.

The purpose of this paper is to develop an Italian adaptation of the Individual Work Performance Questionnaire and test evidence of its validity and reliability. To achieve this goal, the following steps were performed: (1) we compared the mean scores and standard deviation of the Italian and Dutch samples; (2) we first performed an exploratory factor analysis in order to find the same distribution of items in the factors also in the Italian context and then performed a CFA to test the fit indices and confirm the factor structure; thus, we reported the factor loadings of the most parsimonious model and verified the normality of the distribution; (3) we tested for convergent validity; and (4) we tested a series of multiple-group CFAs, in which different and progressively more stringent forms of measurement equivalence (configural, metric, scalar, etc.) were used for the variables gender and occupation type.

## 4. Materials and Methods

### 4.1. Translation Procedure

For the translation procedure from Dutch and English-American to Italian, we followed the recommendations of Beaton et al. [41] through the following three steps: (1) forward translation and adaptation of the original scale from English-American to Italian; (2) back translation; and (3) revision committee. After the original 18-item English-American version of the IWPQ (as presented in the English instruction manual; Koopmans, 2015) [29] was translated to Italian, the first Italian version was retranslated into English-American by a bilingual psychologist with a Ph.D. Once we verified that there were no substantial differences between the final Italian version and the original English-American version, we proceeded with the next step. The revision committee then agreed on the final Italian version.

### 4.2. Participants

This study enrolled a total of 1053 participants (502 men, 47.7%; 551 women, 52.3%); the age of participants ranged between 19 and 69 (M_age_ = 34.6, *SD* = 11.8). Regarding occupation, to carry out a proper comparison with the original version of the scale, we classified the sample following the same way as the Dutch sample in the Italian context, so we have blue collar (manual workers, e.g., carpenter, mechanic, truck driver) at 32.5%, pink collar (service workers, e.g., hairdresser, nurse, teacher) at 33%, and white collar (office workers, e.g., manager, architect, scientist) at 34.5%.

Regarding educational attainment, 37.1% of the sample completed 13 years of schooling (Upper Secondary School), while 62.9% completed a minimum of 16 years of schooling (Bachelor’s, Master’s, and Doctoral Degrees). On average, the participants reported to work 36 h per week (*SD* = 11.9) and have been in the same profession for an average of 14 years (*SD* = 8.6).

It was used as a convenience sampling technique for recruiting companies and participants in this study. Convenience sampling is a non-probability sampling method; the companies and their HR departments were contacted mainly by written correspondence. Employees were thus involved using the company’s communication channels, i.e., social media groups (e.g., LinkedIn), emails, and classic letters of participation. Each participant was sent a cover letter before the questionnaire, explaining the reasons for this research and asking them to answer with complete sincerity. Each participant voluntarily and anonymously took part in this study and read and approved the general objectives of this study and the informed consent before completing the questionnaire. The questionnaire required approximately 15 to 20 min to complete; participants reported at the end that the time was adequate and that they found it easy as far as filling it out was concerned.

### 4.3. Measures

#### 4.3.1. The Individual Work Performance Questionnaire (IWPQ)

For the measurement of the construct of the Individual Work Performance it was used the IWPQ. The scale in its original version consists of 18 items, measuring three distinct factors: task performance, contextual performance, and counterproductive work behavior. As a response mode, all items refer to a 3-month recall period and use a 5-point Likert scale (from ‘rarely’ to ‘always’ for task- and context-related performance, from ‘never’ to ‘often’ for counterproductive work behaviour).

#### 4.3.2. Utrecht Work Engagement Scale (UWES)

For the assessment of the extent of work involvement, the Utrecht Work Engagement Scale was selected, which interprets involvement as a positive and energetic state of mind related to the personal work situation [42,43]. The scale in its full 17-item version measures three different components: vigour, dedication, and absorption [44]. Respondents answered the items on a frequency scale, using a Likert scale ranging from 0 (never) to 6 (always).

#### 4.3.3. Job Satisfaction Survey

The Job Satisfaction Survey developed by Spector [45] (JSS) and validated and adapted in the Italian context by Platania et al. [46] was identified and used to measure job satisfaction. The scale, consisting of 36 items, specifically measures nine different factors, representing nine subscales: Pay, Promotion, Supervision, Fringe Benefits, Contingent Rewards, Operating Conditions, Co-workers, Nature of Work, and Communication.

The scale provides different degrees of satisfaction based on the overall score. The total score is calculated from all items and subscales and can therefore vary from 36 to 216. For the total of the items, the ranges from 36 to 108 indicate dissatisfaction, from 144 to 216 satisfaction, and between 108 and 144 ambivalence.

#### 4.3.4. Stanford Presenteeism Scale (SPS-6)

For the assessment of presenteeism, the Stanford Presenteeism Scale [47] was used. The six-item scale has a five-point Likert scale of agreement as the response mode. The SPS-6 provides a total score that can vary from 6 to 30, and, according to Koopman et al. [47], higher scores indicate a higher level of presenteeism, i.e., a greater ability to concentrate and complete work despite health problems, thus a positive factor.

### 4.4. Data Analysis

To test the psychometric properties of the individual work performance questionnaire in the Italian work context, we used linear structural equation models. All the analyses were performed in AMOS 29.0, applying the maximum likelihood method, which can provide simultaneous estimates of the model parameters. As a first step to verify and confirm the factorial structure of the scale, several confirmatory factor analyses (CFAs) were performed on the data set to identify the best factorial model to fit the data. In addition, different forms of equivalence were tested with a series of multiple-group CFAs performed on the entire sample, grouped by gender and occupation type [48,49]. Configural invariance, metric invariance [50], measurement error invariance [51,52], scalar invariance [53,54], and structural invariance [55,56] were tested.

Several indices were used to verify the goodness of fit of the IWPQ in the Italian context, including the CFI Tucker (the comparative fit index), the RMSEA (the root mean square error of approximation), the G goodness of fit index (GFI), and the standardised root mean square residual (SRMR) to examine the goodness of fit of the model. Values of SRMR close to 0.06 are indicative of a good fit; values between 0.07 and 0.08 are considered a moderate fit; and values between 0.08 and 0.10 are indicative of a marginal fit. For the CFI and GFI indices, higher values demonstrate better adaptation. Values above 0.95 indicate very good adaptation; values between 0.90 and 0.95 indicate marginally acceptable adaptation; and values below 0.90 indicate poor adaptation.

Also used were the χ^2^ and ∆χ^2^ values, presented among the competing models, which assume multivariate normality and are sensitive to sample size.

Akaike Information Criterion (AIC) and Bayesian Information Criterion (BIC) (lower values indicate a better fit) were added to test sensitivity to sample size. The index ∆CFI was also used with values not exceeding 0.01, indicating equivalence of the models in terms of fit [57,58]. Cronbach’s Alpha coefficient [59] was used to test construct reliability at multiple indicators, implemented by the measure of convergent validity tested with average variance extracted (AVE) and construct reliability (CR).

The AVE had to have values >0.50 [60], and the CR had to have values >0.60 [61]. Finally, SPSS 27.0 was used to test correlations.

To optimise the sample size, missing values for relevant items were estimated by the Expectation Maximization method. None of the items had more than 5% missing values, indicating that this option was appropriate [62].

## 5. Results

### 5.1. Descriptive Statistics of the IWPQ Scores: Comparison between the Dutch Sample and the Italian Sample

In Table 1, the mean scores and standard deviation of the three factors of the IWPQ are reported, comparing the two different populations (Dutch and Italian).

As can be seen from Table 1, the averages between the two populations differ significantly in all three factors concerning the IWPQ.

We thus conducted an item analysis to check the goodness of fit of the items in the Italian version. The item analysis showed that by removing one of the items “I complained about minor work-related issues at work” from the counterproductive behaviour factor, the scale had adequate reliability, and the Cronbach’s alpha rose from 0.55 to 0.77.

### 5.2. Convergent Validity

In Table 2, we report the Average Variance Extracted (AVE), the Composite Reliability (CR), the Cronbach’s Alpha, and the correlation matrix for this study variables.

Cronbach’s Alpha was computed for each factor to test reliability and showed good internal consistency of the scale: Task performance 0.75 (in Dutch version was 0.78), Contextual performance 0.88 (in Dutch version was 0.85), and CWB 0.77 (in Dutch version was 0.79). 

Composite reliability and average variance extracted from the IWPQ were CR 0.88, AVE 0.59 for Task Performance, CR 0.89, AVE 0.52 for Contextual Performance, and CR 0.81, AVE 0.52 for CWB.

The high positive relationship between Engagement and Task Performance (r = 0.43, *p* < 0.001) and Contextual Performance (r = 0.42, *p* < 0.001) confirms the same convergent validity of the Dutch study in the Italian context.

Furthermore, as in the Dutch study, engagement correlates negatively with CWB in the Italian study (r = −0.27, *p* < 0.001).

Convergent validity is also confirmed in the Dutch study by the positive correlation between Presenteeism and Task Performance (r = 0.24, *p* < 0.001) and Contextual Performance (r = 0.29, *p* < 0.001) and the negative correlation between presenteeism and CWB (r = −0.19, *p* < 0.001).

Moreover, there is a positive correlation between Job satisfaction and Task Performance (r = 0.32, *p* < 0.001) and Contextual Performance (r = 0.37, *p* < 0.001) and a negative correlation with CWB (r = −0.24, *p* < 0.001) (Table 2).

### 5.3. Exploratory Factor Analysis

In order to verify the underlying theoretical structure of the scale and identify the underlying relationships between measured variables, we carried out an exploratory factorial analysis. Factors with an eigenvalue greater than 1 were retained. Furthermore, within the factors, we retained those items that loaded 0.35 or more than the expected factor [63,64]. This result confirmed the need for changes in the original scale; the item “I complained about minor work-related issues at work”, was eliminated because its internal consistency did not meet the designated adequacy criterion.

We performed a second and third factorial analysis on the remaining items and identified a final corpus of 17 items satisfying the factorial solution.

The percent of the total variance explained was 68.4%. Bartlett’s test of sphericity is significant (χ^2^ (136) = 7991.13, *p* < 0.001), and the test of Kaiser–Meyer–Olkin (KMO = 0.81, BC = 0.849–0.956) indicated a good sampling adequacy of the data. Compared to the original Dutch version in the Italian context, the factorial solution of IWPQ is satisfied by 17 items instead of 18.

### 5.4. Confirmatory Factor Analysis

To test the factorial structure of the IWPQ in the Italian sample in the version of 17 items, we conducted a confirmatory factor analysis by appearing in three different models. A model with three first-order factors with co-variances among them (Model 1) was tested, and the following fit indexes were obtained: [χ^2^(104) = 577.86, SRMR = 0.06, RMSEA = 0.07, CFI = 0.94, GFI = 0.93, AIC = 622.001, BIC = 858.901]; Model 1 was then compared to one second-order factor and three first-order factors (Model 2) [χ^2^(102) = 945.13, SRMR = 0.07, RMSEA = 0.09, CFI = 0.89, GFI = 0.91, AIC = 1047.134, BIC = 1300.063]. Moreover, the first model of the two showed the best fit to the data, based on fit indexes, AIC, BIC, and delta Chi-square value [(Δχ^2^M2-M1(2) = 469.274)]. Model 1 was then compared to a one-factor model (Model 3), in which all the items were predicted by a single factor [χ^2^(105) = 2774.06, SRMR = 0.13, RMSEA = 0.16, CFI = 0.66, GFI = 0.76, AIC = 2870.634, BIC = 3108.685], and it showed again the best fit to the data [(Δχ^2^M3-M1(1) = 2196.2)]. Fit indexes for the tested models are presented in Table 3.

Next, once we identified the most parsimonious model, we reported the items, overall averages with standard deviations, and normality of the distribution in Table 4.

Regarding the normality of the distribution, critical values greater than +2.00 or less than −2.00 indicate statistically significant degrees of nonnormality. The results showed that the data were normally distributed, with acceptable values of skewness and kurtosis. All factor loadings were significant at *p* < 0.001. The results confirm the goodness of scale and normality of the distribution. The list of items in the Italian version is available in Appendix A.

### 5.5. MCFA (Multigroup Confirmatory Factorial Analysis)

As a final psychometric test to verify the goodness and adaptation of the IWPQ in the Italian context, we tested a series of multiple-group CFAs, in which different and progressively more stringent forms of measurement equivalence (configural, metric, scalar, etc.) were used for the variables gender and type of occupation.

#### 5.5.1. Measurement Invariance for Gender

The first multiple-group analysis tested a model of configural invariance (Model 1) by simultaneously evaluating the fit of male and female samples. The fit indices [χ^2^(208) = 932.8, *p* < 0.001; CFI = 0.91; SRMR = 0.072; RMSEA = 0.064] indicated a good fit for this model, supporting an equivalent solution made of three first-order factors with co-variances among them (Table 5). The fit of this configural model provides the baseline value against which all subsequently specified equivalence models are compared [55,56].

Model 2 was tested for metric invariance (Table 5). More importantly, Δχ^2^M2-M1(14) = 38.7 and ΔCFI = 0.001 suggested that Model 2 could be considered equivalent to Model 1. This result indicates that metric invariance is supported.

The third and fourth models tested concerned scalar invariance (Model 3) and error invariance (Model 4) were found (Δχ^2^M3-M2(15) = 73.8, ΔCFI = 0.001; Δχ^2^M4-M3(20) = 44.9, ΔCFI = 0.000).

Also, equivalence in factor variances was tested (Model 5) and confirmed here as valid (Δχ^2^M5-M4(14) = 63.4, ΔCFI = 0.000). Finally, the equivalence in factor covariances was tested (Model 6) by nesting the respective model with Model 5, and the result was that it was supported (Δχ^2^M6-M5(21) = 57.7, ΔCFI = 0.000) (Table 5).

#### 5.5.2. Measurement Invariance for Employment Status

A second multi-group analysis was tested on a configurational invariance model (Model 1) by simultaneously evaluating the fit of the blue collar, pink collar, and white collar.

The fit indices (χ^2^(251) = 978.1, *p* < 0.001; CFI = 0.92; SRMR = 0.068; RMSEA = 0.062) indicated a good fit for this model, supporting an equivalent solution made of three first-order factors with co-variances among them in the data sets for IWPQ in the data sets for blue collar, pink collar, and white collar (Table 6). Model 2 was tested for metric equivalence. Results indicated that Model 2 could be considered equivalent to Model 1, as Δχ^2^M2-M1(12) = 15.3 and ΔCFI = 0.000.

Thus, metric invariance was supported. Model 3 tested for scalar invariance; (Model 3) and error invariance (Model 4) were found (Δχ^2^M3-M2(11) = 156.7, ΔCFI = 0.001; Δχ^2^M4-M3(14) = 109.2, ΔCFI = 0.000).

We then proceeded by testing equivalence in factor variances, and it was found to be supported (Model 5, Δχ^2^M5-M4(17) = 30.05, ΔCFI = 0.000).

Finally, we tested the equivalence in factor covariances (Model 6, Δχ^2^M6-M5(10) = 21.6, ΔCFI = 0.000), and they were both found.

## 6. Discussion

The primary aim of this study was to propose a validated and adapted version of the IWPQ in the Italian context. Indeed, given the importance of measuring this construct and the limited presence of instruments that comprehensively measure the phenomenon, it seemed appropriate to provide an Italian version useful for research and practice. The results suggest that the instrument has very good psychometric properties also in the Italian context, consistent with the other validated version in the European countries [10,38,39,40]. All the items, except for the item “I complained about minor work-related problems”, adequately saturated and supported the factorial structure of the scale in the Italian context. This item was in the Counterproductive Behaviour Factor (CWB), which, from 5 items in the Dutch version, has now become 4 in the Italian version. The CWB factor works better in the Italian version with 4 items.

A possible explanation we can assume for the elimination of this item is of a cultural nature: the respondents indeed disagree with the work environment in these terms based on the way work is conceived in Italy.

In contrast to the original scale, the results of the Italian scale show higher average scores. Although the score was fully adhered to, a possible explanation can be found in the impact that the COVID-19 pandemic had on data collection and the corresponding results: after the pandemic, indeed, the perception concerning the concept of individual performance probably became more acute, as were the demands and resources that the individuals had to put in place and modify.

The concept and paradigm of individual performance itself changed. The pandemic has significantly altered organisational contexts, reducing schedules, changing goals, reducing working hours, or even firing, greatly affecting individual performance [65,66,67]. Company success and profitability are achieved by ensuring that individual energies are linked to company goals, which is why having an adequate and valid measure in the Italian context can help companies to be more competitive and perform better [68].

As to the factorial structure of the scale, a first-order structure is confirmed also in the Italian context; the comparison with the other two models has indeed made it possible to identify and confirm that the best psychometric performance of the IWPQ is given by the impact that each factor has in the description of individual performance. The individual factor loadings are also significant, and reliability is given by the Average Variance Extracted and the Composite Reliability.

Convergent validity also suggests an important and significant correlation with constructs that predict individual performance, such as work engagement, job satisfaction, and presenteeism [46]. These three constructs determine and confirm how individual performance is related not only to work-related operational paradigms but also to the daily relationship the worker establishes with his or her work. It is worth noting that the construct of job performance is also closely related to social capital theory and social exchange theory [23]. In detail, according to social capital theory, social relationships are resources that lead to the development and accumulation of human capital [24]. Employee relations represent the social capital that enables companies to develop. Social exchange theory, on the other hand, suggests that social exchange is limited to actions that are conditioned by reward and gratifying reactions from others [25]. It is a bilateral, mutually contingent, and mutually rewarding process involving ‘transactions’ or simply ‘exchange’. In other words, it means that employees and the organisation can display and enact desired attitudes and behaviours in the workplace [26,27]. Thus, in a sort of positive vicious circle, individuals who are satisfied with the organisation will be able to perform well and adopt extra-role performance occasionally, and similarly, individuals who perform well will also experience higher satisfaction.

Finally, as a further analysis, an invariance by gender and one by occupational status were carried out. The intention here was to examine not only the canonical difference in perceptions of the scale by gender but also to check whether this also occurs for the different occupational categories that we used to categorise our sample, following the indications of the sample and the Dutch study.

This research work still has important limitations that we intend to overcome with future research: this study is cross-sectional in nature, and therefore, in addition to providing partial and non-exhaustive information, such as verifying why the average score is significantly higher than in the Netherlands sample, it is not possible to verify and assess the criterion validity. Criterion validity could provide us with valuable information on the impact of the perception and experience of the concept of individual performance over time, within the same organisational contexts. Despite this, the work has important theoretical and practical implications and can serve as a great support for the Italian organisational community.

## 7. Conclusions

The results, consistent with the original scale, revealed that the Italian version is also invariant for the variables and groups considered.

In conclusion, the IWPQ, even with the elimination of one item, presents an excellent performance that can also be used in the Italian context, and given the scarcity of validated instruments in the Italian context, it may prove to be a useful tool and an excellent indicator of the success and well-being of organisations and the people who work in them. Studies such as this underline the importance of having standardised instruments for measuring important constructs such as the IWP that are valid in different contexts (e.g., Public Administration, Health Care sector) that allow comparisons to be made across countries and can help research and practise learn more about, for example, the important antecedents and consequences of individual job performance [69,70,71,72].

Adequate studies on the importance of individual performance can be used to better understand and distinguish the different components affecting performance. For example, Rotundo and Sackett [31] have an interesting overview relating to the literature review that helps to understand how all the components that concern work performance are fundamental and that they are linked to task, citizenship, and counterproductive performance. They also point out that, even if the task is counterproductive and the performance is dominant, the extent to which they do so depends on who provides the evaluation.

In addition, it is important to link individual performance to other variables that may affect its effect and duration, such as happiness, project management, organisational and safety climate, leadership, etc. [73,74,75,76,77,78,79].

## Figures and Tables

**Table 1:** The mean (M) and standard deviation (SD) of the IWPQ scores were compared between the Dutch sample and the Italian sample.

	Dutch Sample (N = 1424)			Italian Sample (N = 1053)		
	Task Performance	Contextual Performance	CWB	Task Performance	Contextual Performance	CWB
	M (SD)	M (SD)	M (SD)	M (SD)	M (SD)	M (SD)
**Blue collar**	2.77 (0.62)	2.30 (0.82)	1.03 (0.63)	3.42 (0.71)	3.29 (0.87)	3.39 (1.05)
**Pink collar**	2.68 (0.63)	2.31 (0.76)	1.09 (0.71)	3.36 (0.74)	3.33 (0.88)	3.45 (1.08)
**White collar**	2.55 (0.63)	2.34 (0.72)	1.21 (0.66)	3.35 (0.70)	3.30 (0.91)	3.43 (1.06)
**Total sample**	2.67 (0.63)	2.31 (0.77	1.11 (0.67)	3.38 (0.72)	3.31 (0.89)	3.42 (1.06)

**Table 2:** Convergent validity.

	α	CR	AVE	1	2	3	4	5
Task Performance	0.75	0.88	0.59	1				
2. Contextual Performance	0.88	0.89	0.52	0.38 **	1			
3. CWB	0.77	0.81	0.52	−0.22 **	−0.25 **	1		
4. Work Engagement	0.92	0.91	0.65	0.43 **	0.42 **	−0.27 **	1	
5. Job satisfaction	0.89	0.87	0.57	0.32 **	0.37 **	−0.24 **	0.52 **	1
6. Presenteism	0.88	0.85	0.55	0.24 **	0.29 **	−0.19 **	0.39 **	0.26 **

Note: ** *p* < 0.001.

**Table 3:** Fit indexes for models tested in CFA.

	χ^2^	df	SRMR	RMSEA	RMSEA 90%-C.I.	CFI	GFI	AIC	BIC
Model 1 ^a^	577.86 *	104	0.06	0.069	0.067–0.075	0.94	0.93	622.001	858.901
Model 2 ^b^	945.13 *	102	0.07	0.089	0.084–0.094	0.89	0.91	1047.134	1300.063
Model 3 ^c^	2774.06 *	105	0.13	0.157	0.150–0.160	0.66	0.76	2870.634	3108.685

Note: ^a^ Model 1: three first-order factors with co-variances among them; ^b^ Model 2: one second-order factor and three first-order factors; ^c^ Model 3: all the items were predicted by a single factor; CFI = Comparative Fit Index; GFI = Goodness of Fit Index; AIC = Akaike Information Criterion; RMSEA = Root Mean Square Error of Approximation; SRMR = Standardized Root Mean Square Residual; BIC = Bayesian Information Criterion; * *p* < 0.05 level.

**Table 4:** Descriptive statistic, normality of distribution, and factor loading of model 1.

In the Past 3 Months	M	SD	Skewness	Kurtosis	Factor Loading Model 1
I was able to plan my work so that I finished it on time (TP).	3.32	1.24	1.80	0.69	0.663
2. I kept in mind the work result I needed to achieve (TP).	3.36	0.92	0.85	0.71	0.754
3. I was able to set priorities (TP).	3.36	1.10	1.61	1.04	0.836
4. I was able to carry out my work efficiently (TP).	3.48	0.88	1.28	1.59	0.855
5. I managed my time well (TP).	3.36	1.17	1.84	1.13	0.707
6. On my own initiative, I started new tasks when my old tasks were completed (CP).	2.83	1.12	−0.99	0.42	0.733
7. I took on challenging tasks when they were available (CP).	2.99	0.99	−1.01	1.07	0.833
8. I worked on keeping my job-related knowledge up-to-date (CP).	3.12	0.99	−0.95	0.27	0.695
9. I worked on keeping my work skills up-to-date (CP).	3.19	0.99	−1.14	0.54	0.641
10. I came up with creative solutions for new problems (CP).	2.94	1.03	−0.83	0.16	0.759
11. I took on extra responsibilities (CP).	2.57	1.25	−0.60	−0.56	0.687
12. I continually sought new challenges in my work (CP).	2.73	1.14	−0.60	−0.41	0.798
13. I actively participated in meetings and/or consultations (CP).	2.85	1.21	−1.04	−0.08	0.569
14. I made problems at work bigger than they were (CWB).	3.47	0.99	−0.76	3.43	0.717
15. I focused on the negative aspects of situation at work instead of the positive aspects (CWB).	2.96	1.15	−0.13	−0.48	0.666
16. I talked to colleagues about the negative aspects of my work (CWB).	2.22	1.251	−0.881	−1.07	0.786
17. I talked to people outside the organization about the negative aspects of my work (CWB).	2.97	1.212	−0.888	−0.427	0.714

**Table 5:** Fit statistics for measurement invariance by gender.

Model	χ^2^ (df)	CFI	SRMR	RMSEA	ΔCFI
1. Configural Invariance	932.8 (208)	0.91	0.07	0.06 (0.066–0.072)	-
2. Metric Invariance	971.5 (222)	0.90	0.08	0.06 (0.062–0.071)	0.001
3. Scalar Invariance	1045.3 (237)	0.89	0.08	0.06 (0.062–0.071)	0.001
4. Measurement error Invariance	1090.2 (257)	0.89	0.08	0.06 (0.061–0.069)	0.000
5. Structural Variance Invariance	1153.6 (271)	0.89	0.08	0.06 (0.061–0.069)	0.000
6. Structural Covariance Invariance	1211.3 (292)	0.89	0.08	0.06 (0.061–0.069)	0.000

**Table 6:** Fit statistics for measurement invariance by employment status.

Model	χ^2^ (df)	CFI	SRMR	RMSEA	ΔCFI
1. Configural Invariance	978.1 (251)	0.92	0.07	0.06 (0.058–0.073)	-
2. Metric Invariance	993.4 (263)	0.92	0.07	0.06 (0.051–0.069)	0.000
3. Scalar Invariance	1150.1 (274)	0.91	0.06	0.06 (0.051–0.069)	0.001
4. Measurement error Invariance	1259.3 (288)	0.91	0.06	0.06 (0.051–0.069)	0.000
5. Structural Variance Invariance	1289.8 (305)	0.91	0.06	0.06 (0.051–0.069)	0.000
6. Structural Covariance Invariance	1311.4 (315)	0.91	0.06	0.06 (0.051–0.069)	0.000

## Data Availability

The data presented in this study are available on request from the corresponding author. The data are not publicly available due to privacy.

## Appendix A. List of the Items in Italian, Version 17-Item

**Task performance (5 items)**	
**Negli ultimi 3 mesi …**	
**1.**	Sono stato in grado di pianificare il mio lavoro in modo da finirlo in tempo.
**2.**	Ho tenuto presente l’obiettivo da raggiungere.
**3.**	Sono stato in grado di determinare le priorità.
**4.**	Sono stato in grado di svolgere il mio lavoro in modo efficiente.
**5.**	Ho gestito bene il mio tempo.
**Contextual performance (8 items)**	
**Negli ultimi 3 mesi …**	
**6.**	Di mia iniziativa, ho iniziato nuovi compiti quando ho completato i precedenti.
**7.**	Ho intrapreso compiti impegnativi quando erano possibili.
**8.**	Ho lavorato per mantenere le mie conoscenze sul lavoro sempre aggiornate.
**9.**	Ho lavorato per mantenere le mie competenze di lavoro aggiornate.
**10.**	Ho pensato a soluzioni creative per risolvere i problemi.
**11.**	Ho preso ingenti responsabilità.
**12.**	Ho cercato continuamente nuove sfide nel mio lavoro.
**13.**	Ho partecipato attivamente alle riunioni e/o alle consultazioni.
**Counterproductive work behaviour (4 items)**	
**Negli ultimi 3 mesi …**	
**14.**	Ho creato problemi al lavoro più grandi di quanto fossero.
**15.**	Mi sono concentrato sugli aspetti negativi della situazione sul lavoro invece degli aspetti positivi.
**16.**	Ho parlato con i colleghi degli aspetti negativi del mio lavoro
**17.**	Ho parlato con la gente esterna all’organizzazione degli aspetti negativi del mio lavoro.
